# Seeds with low phosphorus content: not so bad after all?

**DOI:** 10.1093/jxb/ery313

**Published:** 2018-10-12

**Authors:** Doris Vetterlein, Mika Tarkka

**Affiliations:** 1 Helmholtz Centre for Environmental Research UFZ, Halle, Germany; 2 Martin-Luther-University Halle-Wittenberg, Halle, Germany; 3 German Centre for Integrative Biodiversity Research (iDiv) Halle – Jena – Leipzig, Leipzig, Germany

**Keywords:** Cereals, imaging, phosphorus, rhizosphere, root age/ontogeny, seedling establishment, seeds, temporal and spatial resolution

## Abstract

This article comments on:

Julia CC, Rose TJ, Pariasca-Tanaka J, Wissuwa M. 2018. Phosphorus uptake commences at the earliest stages of seedling development in rice. Journal of Experimental Botany 69, 5233–5240.

**Phosphorus is of prime importance for growth and vigour during early plant development, hence high seed P content has been regarded as essential for seedling establishment. Julia *et al.* (2018) challenge this dogma, showing the onset of P uptake by seedlings as early as 3 days after germination and that this is unaffected by seed P concentration. Even more important is that the abundance or expression of P transporter genes in roots is not delayed until seed P reserves have been mobilized. This suggests that breeding for low seed P content could be used as a means of reducing the P requirement of cereals in general.**

Plant uptake and translocation of available Pi is mediated by a selective and active system of membrane-spanning phosphate transporters (PTs), which are grouped, based on sequence identity and their localization patterns, into five families. Biochemical analyses indicate that members of PT family 1, which were investigated at the gene expression level by Julia *et al.* (2018), are mostly high-affinity transporters: they contribute to Pi acquisition from soil at limiting, micromolar concentrations. In rice, PT family 1 includes 13 members (Paszkowski *et al.*, 2002). Most of these 13 PT genes are under the control of the phosphate starvation response transcription factors, central regulators of Pi starvation signalling, and their expression is stimulated by low available P (Gu *et al.*, 2016). However, some PT genes are inducible by arbuscular mycorrhizas (AM) and, in symbiosis, the AM Pi uptake pathway might even dominate root Pi uptake (Bucher, 2007). Furthermore, transcript abundances of other PT genes are modulated by plant hormone levels, suggesting that the regulation of P homeostasis is associated with ongoing crosstalk with hormonal signalling (Liu *et al.*, 2011) and by the levels of other nutrients (Gu *et al.*, 2016).

Julia *et al.* showed that the uptake capacity of roots (Pi uptake/root biomass) is higher when the seeds have a low starting P concentration, suggesting that a Pi sensor system governs the maintenance of Pi homeostasis. In rice and Arabidopsis, the SPX1 protein has been characterized as an intracellular Pi sensor that interacts with PHR1 and regulates plant Pi concentration. On the other hand, inhibiting *OsPT2* or *OsPT6* expression by RNA interference significantly decreased both the uptake and the long-distance transport of Pi from roots to shoots (Ai *et al.*, 2009), suggesting that some of the rice PTs are also involved in the regulation of Pi homeostasis. Since Julia *et al.* observed that *OsPT* transcript abundances were not modified by seed P levels, it is possible that the activity of the transporters is regulated at the protein level. Interestingly, post-translational modifications are widespread among members of PT family 1 and involve protein–protein interactions, ubiquitination and phosphorylation affecting the localization, abundance and activity of the PTs (Gu *et al.*, 2016; Wang *et al.*, 2017).

The authors also showed that whereas transcripts of rice P transporter genes *OsPT1*, *OsPT2, OsPT4* and *OsPT8* were detectable in roots of young seedlings, *OsPT6* and *OsPT9* expression was confined to older roots. This indicates that root age *per se* influences transporter expression. The observation adds a new layer of knowledge about P transporter gene expression. Previous work has mostly focused on tissue and cell type-related patterns of transcript abundances. For instance, OsPT2 is localized exclusively to the stele of primary and lateral roots whereas OsPT6 is present in both epidermal and cortical cells of the younger primary and lateral roots (Ai *et al.*, 2009). Future research could focus on the regulation of transporter expression during root system development in more detail. Open questions are whether the developmental regulation of the transporters can be modified by external (P, other elements, water) or internal (plant hormonal status) drivers, and the relative importance of PT regulation at gene expression and protein levels.

## Modifying the root system surface to increase Pi uptake

The observations of Julia *et al.* (2018) suggest that the total amount of acquired Pi may be limited by root size in slow-growing rice genotypes. This indicates that genotypes with more-rapid root development acquire more total P from soil solution. To improve soil exploration at P deficiency, plants do commonly modify their root system architecture and root hair density (Péret *et al.*, 2011; Nestler *et al.*, 2016). For instance, this may be through an increase in root surface area by enhanced formation of fine roots and root hairs, suppression of primary root elongation but proliferation of lateral roots, or, as has been well described in rice, increased root elongation (Wang *et al.*, 2017). This suggests that genotypes with faster development of the root system may more rapidly add to the P reserves from the seeds. Data from P starvation-related transcription factors in rice underline the importance of root system architecture in Pi acquisition. Not only do the transcription factor activities alter the expression of several Pi transporters, but also the extents of root elongation and root hair formation (Gu *et al.*, 2016; Dai *et al.*, 2016).

Root development and exudation patterns are further modified by members of the rhizosphere microbiota including bacteria, and endophytic and mycorrhizal fungi. The so-called co-operation in the rhizosphere (Barea *et al.*, 2005) takes place in Pi acquisition, and is based on enhanced phosphate solubilization, transport by AM fungi and plant roots, and plant growth promotion. In rice field trials, such microorganisms have been used to increase fertilizer use efficiency (Bakhshandeh *et al.*, 2015). Now, in the context of the report by Julia *et al.*, it would be interesting to know how early-stage rice roots interact with the rhizosphere microbiome to facilitate efficient Pi uptake.

## Moving into the soil

The observations of Nestler *et al.* (2016) suggest that the use of natural substrate is a prerequisite for estimating the impact of P on root development in rice. In upland field soil, rice roots produced short root hairs, and their abundance was not affected by P deficiency. Under these conditions, higher root hair density occurred at high available P, which was in stark contrast to the plants grown in nutrient solution: they produced more and longer root hairs in low P conditions. In order to obtain a holistic view of P allocation in plant tissues and uptake of available Pi from soil, non-invasive means of analysis should be integrated into a detailed analysis of the plant–soil system, including both space and time as variables. Localization of transporter gene expression activities to different tissues and areas of the root system at different ages, and measures of Pi uptake, should be combined with the analysis of transporter abundances.

## Relevance of early P uptake activity

The temporal development of P uptake capacity and its relation to seed P is not only important for breeding but highly relevant for the increasing number of imaging approaches in rhizosphere research, whether they relate to molecular biology, microbiology, chemistry or physics (Roose *et al.*, 2016). New techniques enable investigations in soil *in situ*, partly non-destructively, so avoiding artefacts caused by altered water or gas availability along transparent plates. However, most imaging approaches, which are conducted to reveal parameter patterns and improve our understanding of small-scale processes, are restricted to early growth stages: many techniques show a trade-off between size of the object and image resolution (CT, MRI, neutron radiography), and moreover spatial gradients, addressed by imaging, are obscured once pot size alters root system geometry (Blaser *et al.*, 2018).

As long as approaches have been based on root mats or more generally on delimiting root systems from the bulk soil by meshes/membranes, results relate to a mix of roots showing different age (Roose *et al.*, 2016). New approaches, with their small field of view, enable investigation of individual roots with their surrounding soil with very high spatial resolution (Vetterlein *et al.*, 2018). Thus, the location needs to be selected carefully and more detailed information about spatial and temporal distribution of root activity is required. The onset of uptake activity after germination as reported by Julia *et al.* is just one important stepping stone (see Box 1). We are still missing information on how long a root segment remains active and whether the information observed for the 3-cm long seminal roots of rice can be transferred to all root segments of that age, even though the plant, as a whole, is older. Julia *et al.* have shown that actual uptake and depletion of the exogenous P coincided with the expression of membrane transporter genes, which basically allows selection of either of the two approaches for future studies.

**Box 1. Relevant parameters for the formation of nutrient gradients in the rhizosphere**

Uptake dynamics of a root segment are potentially regulated by exogenous and endogenous nutrient availability as well as the developmental stage of the root segment in question. All parameters show distinct dynamics in space and time which can be modulated by environmental conditions. As an example, 3D-root age distribution was depicted for a 16 day old *Vicia faba* plant grown in a soil-based column experiment. Underlying the root system is a distance map illustrating the travel distance for nutrients to the closest root surface for each soil voxel assuming homogenous nutrient distribution. Thanks to S. Schlüter and S. Blaser for analysis; further details provided by Blaser *et al.* (2018), Schlüter *et al.* (2018).

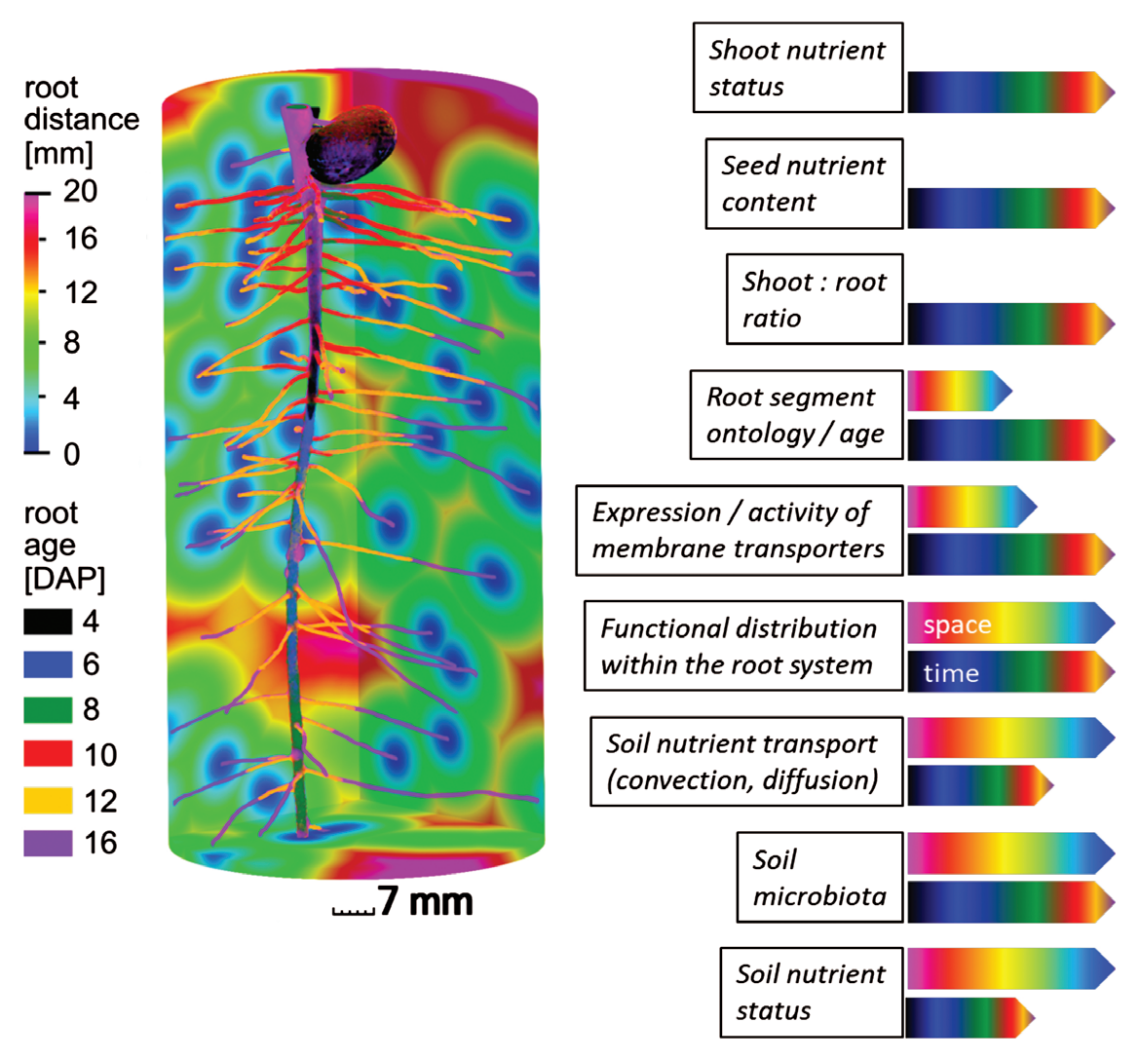

## Stepping forward

For high resolution approaches, it might be possible to combine tissue- or cell-specific gene expression (Yamaji and Ma, 2011; Yu *et al.*, 2016) with measurement of spatial gradients extending into the soil (Roose *et al.*, 2016). Even more tempting would be to combine the imaging of chemical, microbiological or physical gradients with the imaging of gene expression or transporter activity – an attempt which is at the core of the new priority programme (PP 2089) of the German Research Foundation (http://www.ufz.de/spp-rhizosphere/). Requirements for sampling and sample preparation currently differ widely between the different techniques, hindering measurement of different parameters for one and the same root segment. Root age as a proxy for root development (ontogeny) could serve as a common reference for data synthesis (Vetterlein and Doussan, 2016). Root age can be determined in soil *in situ* with X-ray CT (Koebernick *et al.*, 2015) as well as in nutrient solution studies. Based on root segment age, spatiotemporal functional diversity can be included in 3D models on root water and nutrient uptake from heterogeneous soil environments (Dunbabin *et al.*, 2013).
